# Making Hard Choices in Local Public Health Spending With a Cost-Benefit Analysis Approach

**DOI:** 10.3389/fpubh.2019.00147

**Published:** 2019-06-28

**Authors:** Lee Robertson, Chris Skelly, David Phillips

**Affiliations:** Public Health Dorset, Dorchester, United Kingdom

**Keywords:** economic model, decision–making, cost benefit analyses, public health, health ecomic perspectives

## Abstract

**Background:** In 2013, public health moved into Local Authorities, but initial optimism has been overtaken by serious ongoing financial constraints and an uncertain future. Hard choices have become an everyday reality across local authorities and for their public health teams. Assessing the return-on-investment of public health interventions and possessing economic evaluation skills have become more critical than ever before.

**Methods:** Using the New Economy cost-benefits-analysis model developed at the Greater Manchester Combined Authority, we undertook cost benefit analyses of some of our largest areas of commissioned spend in local public health practice to better understand both the public and fiscal returns of our interventions.

**Results:** The cost-benefit analyses indicated considerable variation in the public (economic and social) returns-on-investment for our spend on services purchased as a commissioner with £1.37 to 6.81 returned for every £1 spent, and a fiscal return for every £1 invested of between £0.54 and 1.37. Additionally, the fiscal benefits (reduced service costs) of these public health interventions appear to primarily flow to the NHS, which accounts for about 94% of the fiscal return.

**Conclusion:** While cost-benefit modeling cannot provide a complete picture of “value,” it does provide decision-makers with a transparent metric that facilitates a whole-of system discussion on “intervention value” and prevention at scale investments. This approach will support investment strategies when implementing Sustainability and Transformation Partnerships and Integrated Care Systems. However, these tools should be used to support robust decision-making processes, not as a replacement for or a short-circuiting of existing processes.

## Introduction

Scarcity and austerity have become the “new norm” in the UK public service and this is very apparent in the funding of local public health services (1, 2). The transfer of public health monies and responsibilities from the NHS to Local Authorities in 2013 was seen by many as a positive step. Notions of “public health returning home” and the like abounded. Mandated programmes and ring-fenced grants reinforced notions of a more secure future.

However, this initial optimism has been overtaken by harsh local and national financial realities with significant reductions to both local authority general funding and to the national public health grant over the period 2015–2020 and with a very uncertain future beyond that.

Against this backdrop and that of increasing need, inequality and poverty (3, 4), hard choices have become an everyday reality for public health teams. Nationally and locally assessing the return-on-investment of public health interventions and possessing skills in the use of economic evaluation methods for informed cost-benefit and cost-effectiveness decision-making have become more critical than ever before (5).

A WHO report, *The Case for Investing in Public Health*, highlights cost-effective interventions that provide returns on investment and/or cost savings in either the short or the longer term (6), while National Institute for Health and Care Excellence (NICE) has broadened its approach to the appraisal of public health interventions (7). Local authorities are responsible not only for the health of individuals and communities, but also for their overall welfare. The tools of economic evaluation must reflect a wider remit than health and have a greater local element within them (7).

A change of approach to economic evaluation by NICE in recent times has placed more emphasis on cost–consequences analysis (CCA) and cost–benefit analysis (CBA) than has been the case in previously. Cost-effectiveness analysis (CEA) and cost–utility analysis (CUA) will still be required for several reasons: CEA and CUA provide a single “currency” for measuring the impact of interventions on health and it allows comparisons of interventions in healthcare to allow the efficient allocation of resources (7).

This paper estimates the return-on-investment (ROI) for public health activities across Dorset, Bournemouth and Poole during the financial year of 2016/17. We identify the amount of work done and the cost associated with five main commissioned programmes, Drugs and Alcohol, LiveWell Dorset behavior changes services, the nationally mandated Health Checks, Smokestop and Sexual Health, as well as the benefits potentially arising from this effort.

## Materials and Methods

### Cost-Benefit Analysis

**Figure 1:**
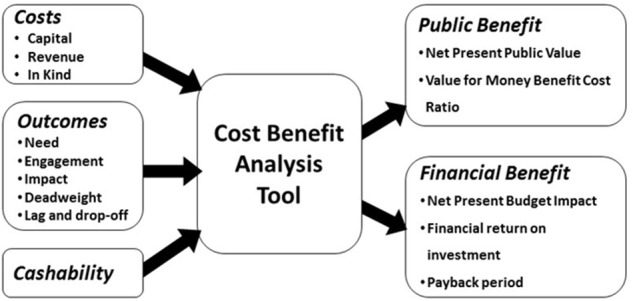
New Economy CBA tool.

CBA provides a universal metric to support decision-making that allocates scarce resources, both at the fundamental level of where to invest and in the marginal analysis of how much to invest. New Economy's CBA model produces two types of estimated benefit: fiscal benefit and public benefit (Figure 1). Fiscal benefit is the estimated direct savings to other public service programmes resulting in reduced expenditure and public benefit is the wider economic and social returns from a programme (e.g., economic growth, improved health, and well-being).

CBA requires an estimation of both the cost of programmes and the perceived monetary value of the benefit. While programme costs can often be challenging to estimate accurately, it is the estimation of monetary benefit that often proves more difficult.

We estimate monetary benefit as follows:

$$\begin{array}{l} {\text{Pt}}_{\;}\times \;{\text{Pr}}_{\;}\times \text{ Pc }\times \;\left(\text{Ps}-\text{Pd}\right)\;\times \text{ Mv }\times \;\left(\frac{\text{Ob}}{100}\right) \end{array}$$

Where,

Pt (Population target)—number of individuals who could benefit

Pr (Population reach)—proportion of Pt who are offered an intervention

Pc (Population compliance)—proportion of Pr who undergo an intervention

Ps (Population successful)—proportion of Pc for whom the intervention is successful

Pd (Population deadweight)—proportion of Ps who would have achieved the same benefit in the absence of the intervention (i.e., business as usual scenario)

Mv (Monetary value)—the per person benefit (£) generated by the intervention

Ob (Optimism bias)—correction factor to account for data quality of model inputs

The challenge is clearly around the assumptions made, especially the sensitivity of these estimated benefits to changes in these assumptions, which arise from natural variation and uncertainty derived from a lack of knowledge. Five of the assumptions revolve around estimating whom a programme of work affects (Pt, Pr, Pc, Ps, and Pd) and how we value the impact on these individuals (Mv).

Monetary value (Mv) assumptions are particularly controversial. Consequently, the CBA tool used in our work here is designed to support public service transformation analysis in local authorities (5). Therefore, CBA used in this analysis uses existing guidance on Mv from HM Treasury's Green Book (8), which provides consistent, well-researched and conservative estimates.

Finally, the CBA tool applies an optimism bias (Ob) correction factor in response to the level of uncertainty in the data or assumptions used, with a confidence range from 0% (no perceived bias) to −40% (assumptions likely to be very optimistic). This is determined by confidence in the data source, the age of data, and known data error following consultation with programme stakeholders (Table 1).

**Table 1:** Optimism bias (Ob) parameter used in CBA modeling (7) (New Economy 2016).

**Confidence grade**	**Color coding**	**Population/Cohort data**	**Evidence base (engagement/impact)**	**Age of data/analysis**	**Known data eor**		**Optimism bias connection**
1		Figures taken from agency data systems	Randomized control trial in UK	Current data (<1 year old)	±2%	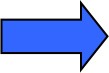	0%
2		Figures derived from local stats	International randomized control trial	1–2 years old	±5%	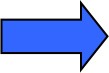	−5%
3		Figures based on national analysis in similar areas	Independent monitoring of outcomes with a robust evaluation plan	2–3 years old	±1 0%	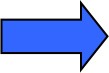	−10%
4		Figures based on generic national analysis	Practitioner monitoring of outcomes with a robust evaluation plan	3–4 years old	±1 5%	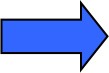	−15%
5		Figures based on international analysis	Secondary evidence from a similar type of intervention	4–5 years old	±20%	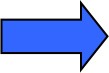	−25%
6		Uncorroborated expert judgment	Uncorroborated expert judgment	>5 years old	±25%	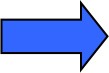	−40%

The CBA tool used in our analysis is implemented in a spreadsheet and takes into account the importance of (5):

- Agreeing the objectives of an intervention, the indicators to measure achievement and the timescale over which outputs and outcomes will be monitored
- Fiscal impact which is the net cost to the public sector of the programme
- Public value which includes all calculable elements of public welfare including economic and social benefits
- Identifying difference between costs and benefits counted in public benefit and those that may be counted in fiscal benefit
- Counting all the costs incurred to deliver a programme
- Applying a discount rate to costs and benefits realized in future years
- Adjusting CBA outputs to account for risk and uncertainty

A final assumption, is the concept of “cashability,” which refers to the extent to which a change in an outcome or an improvement in the way these outcomes are achieved (e.g., process efficiencies) results in a reduction in fiscal expenditure. These are the savings that are available to be reallocated to other programmes. CBA, therefore, informs discussions around how far benefits are cashable; it is not a substitute for not having negotiation between different stakeholders.

### Quantifying Return on Investment

Both public and fiscal benefit are most often represented as a monetary benefit value (£) or as a ratio of the returned monetary benefit to the cost (e.g., 2:1, a return of £2 for every £1 invested). However, an additional measure termed the Public Value for Money (PVM) links the public and fiscal benefits found in the HM Treasury Green Book (8), providing an overall return on investment ratio for a given level of spending.

$$\begin{array}{l} \left(\text{PVM}\right)\;=\;\frac{\text{public benefit}-\text{costs}}{\text{costs}-\text{fiscal benefit}} \end{array}$$

Quantification of return on investment is usually undertaken over a time period of many years, therefore net present public value (NPV) is used to account for the change in value of monetary units over time. Consequently, these costs and benefits are discounted. NPV discounts the estimated benefits and costs of programmes over the probable lifetime of the programme.

$$\begin{array}{l} \text{NPV}\left(\text{i},\text{ N}\right)\;\overset{\text{N}}{\underset{\text{t}=0}{\sum }}\;\frac{{\text{R}}_{\text{t}}}{\left(1+\text{i}\right){\;}^{\text{t}}} \end{array}$$

Where

*t* = the unit of analysis (usually a year)

*i* = the discount rate (3.5%)

*R*_*t*_ = the value of the public benefits—costs (£)

*N* = lifetime of the intervention in units of analysis (number of years)

NPV is the discounted value of a stream of future costs or benefits. It is used to describe the difference between the present value of a stream of costs and a stream of benefits. A discount rate must be applied when calculating an NPV to convert all costs and benefits to “present values.” The Treasury's recommended discount rate is 3.5% (8).

Where programmes run for many years, the Payback Period (PP) illustrates how quickly a return on an investment occurs (to plan where to reinvest savings elsewhere at a certain point in the future). The payback period is:

$$\begin{array}{l} \text{PP }=\;\left(\text{p }-\text{ n}\right)\;÷\text{ p }+\;{\text{n}}_{\text{y}} \end{array}$$

Where,

p = number of years into the future where the first positive cumulative savings exceeds cumulative costs

n = number of years into the future where the last negative value of cumulative costs exceed savings

ny = number of years after initial investment where the last negative present value of cumulative savings occurs.

### Public Health Programmes

We examine 13 outcome areas across five commissioned public health programmes: Drug and alcohol, Health checks, Smoke stop, Sexual health, LiveWell Dorset, and identified total costs over the 2015/16 financial year (Tables 2–4):

Sexual Health Services (£7.2 M): Includes contraceptive, sexual health and asymptomatic screening. Outcomes include chlamydia rates in young people, under 18 teenage conception rates and late diagnosis of HIV.
Drug and Alcohol Services (£4.5 M): Includes alcohol and drug dependency reduction programmes. Outcomes include treatment of alcohol, opiate and non-opiate users with the aim of reducing dependency.
Smokestop Programme (£0.8 M): Smokestop offers six sessions with trained advisors who talk through all aspects of giving up smoking. Outcomes include number of smokers who quit.
Health Checks Programme (£0.7 M): Personalized advice from a doctor or pharmacist on reducing the potential risk of cardiovascular disease. Outcomes include the number of Health Checks completed, the number of prescriptions of statins made, diagnosis of diabetes, and prescriptions of hypertension drugs.
LiveWell Dorset (£0.8 M): Support service for adults who would like support to change their lifestyle. It offers health and well-being information, advice and support. Outcomes include stopping smoking, increasing physical activity, weight management, and reducing alcohol intake.

**Table 2:** Commissioned public health service costs (2015/16).

	**2015/16 (£)**
Sexual health	7,198,472
Contraception	2,411,835
Testing and treatment	4,467,903
Advice and prevention	318,734
Drug and alcohol	4,500,000
Substance misuse	2,470,284
Alcohol misuse	47,848
Drug prevention programme	64,140
Dorset smokestop	778,115
Healthchecks	650,414
LiveWell dorset	750,000
Total	£13,877,001

**Table 3:** Commissioned public health service population parameters (2015/16).

**Programme**	**Outcomes**	**Target population**	**Reach pop**.	**Reach %**	**Engagement %**	**Impact %**	**Deadweight %**
Sexual health	Reducing STI	86,982	18,816	22	100	92	5
	HIV testing	15,561	12,880	83	100	5	5
	Teenage pregnancy	12.514	357	3	100	64	5
Drugs and alcohol	Reduced opiate dependency	3,812	1,944	51	86	9	5
	Reduced alcohol dependency	8,043	1,046	13	85	40	5
	Reduced Alcohol and non-opiates	2,150	430	20	20	77	5
	Reduced Non-opiates only	572	218	38	38	75	5
Dorset SmokeStop	Reduced smoking	94,000	11,124	8	61	53	5
Health check	People requiring statins	45,252	45,252	30	17	11	10
	People at risk with diabetes	45,252	45,252	30	17	11	10
LiveWell dorset	Reduced weight	367,900	4,617	1	42	58	5
	Reduced smoking	94,000	1,621	2	17	19	5
	Increased physical activity	128.400	1,230	1	58	31	5
	Reduced alcohol consumption	129,900	296	<1	57	28	5

**Table 4:** Monetary value (Mv) assumptions in commissioned public health service CBA.

**Outcomes**	**Fiscal benefit (£)**	**Public Benefit (£)**				**Unit cost notes/assumptions**
		**Fiscal**	**Economic**	**Social**	**Total**	
Reduced drug dependency	£3,614	£3,614	£8,954	£3,814	£16,382	Crime reduction benefits of drug treatment and recovery (National Treatment Agency for Substance Misuse, 2012), p.11 (9); and Drug Treatment Outcomes Research Study (10), p.13
Reduced weight	£100	£100	£32	£16	£148	£100 average cost of weight management programmes (11). (source: PHE)
Reduced smoking	£200	£200	£765	£765	£1,730	£200 one to one support (NICE ROI Tool) (12) ASH Reckoner Tool cost of smoking to society per year £1,730 per person (13)
Increased Physical activity	£10	£10	£368	£74	£452	£10 one to one brief advice (Physical activity ROI Tool NICE). £368 workplace interventions (NICE) £74 (Health ROI Physical activity tool) (14)
Reduced alcohol dependency	£1,800	£1,800		£1,398	£3,198	NICE Clinical Practice Guidance 115. £1,800 incl cost of hospital inpatient and day, outpatient, A&E and ambulance, primary care consultations and prescribed medications (for higher risk drinkers). Social value – health/well-being impacts on individual entering treatment. Sourced from analysis of brief interventions delivered in GP surgeries (15)
People requiring statins	£95	£95		£1,772	£1,867	NHS Ready Reckoner savings over 10 yrs. (16)
People at risk with diabetes	£218	£218		£1,772	£1,990	NHS Ready Reckoner savings over 10 yrs. (16)
Reducing high blood pressure	£135	£135		£3,306	£3,441	NHS Ready Reckoner savings over 10 yrs. (16)
Reducing STI	£131	£131		£370	£501	Public Health England. £131–routine STI treatment appointment including chlamydia screening and condom promotion scheme (17). Screening and treatment to reduce chlamydia: £370 per QALY gained (18)
HIV testing	£7,000	£7,000			£7,000	Public Health England. £280k−360k–estimated cost of a lifetime of treatment for an individual infected with HIV borne by the NHS (19)
Teenage pregnancy	£1,207	£1,207	£13,277		£14,484	Public Health England. Cost of untended pregnancy = £1,207 (20). Every £1 spent on prevention generates £11 of benefits (21)

There are many limitations in assessing these programmes. It is often difficult to precisely match a specific cost to a specific outcome due to the way it has been recorded in the financial system. The estimation of cashable savings from any programme is going to be imprecise: Estimates of what is cashable will be approximate and based on negotiations between commissioners and providers rather than solely on a formula or calculation. Nonetheless, we had to make an estimate for the cashability of these savings for our outcome areas (Table 5).

**Table 5:** Relevant cashability assumptions for known programme relationships.

**Commissioned programmes**	**Description**	**Local authority**	**NHS**	**Police**	**Probation**	**Courts/Legal aid**	**Prisons**	**Other CJS**	**DWP**	**Schools**
Drug and alcohol	Reduced drug dependency		59%	19%	2%	7%	9%	5%		
Drug and alcohol	Reduced alcohol dependency		100%							
Health checks	People requiring statins	20%	80%							
Health checks	People at risk with diabetes	20%	80%							
Health checks	Reducing high blood pressure	20%	80%							
Live well	Reduced weight		80%						20%	
Live well	Reduced smoking	20%	80%							
Live well	Increased Physical activity	20%	50%						30%	
Live well	Reduced alcohol consumption		100%							
Sexual health	Reducing STI		100%							
Sexual health	HIV testing		100%							
Sexual health	Teenage pregnancy	10%	60%						20%	10%
Smoke stop	Reduced smoking–smokestop	20%	80%							

Commissioned services such as Health Visitors, School Nursing, environmental health interventions, and our work with partners to embed population health considerations in the policies and actions of other public services have not yet been modeled, because it is much more difficult to link programme costs with specific outcomes. This work requires greater specification of what the intervention is and how we expect it to provide impact.

## Results

### Public Return on Investment

Public Health Dorset spent approximately £12 M in 2015/16 on five commissioned public health service areas. Over a 10-year period, these activities generate an estimated £30 M in public benefit, of which the majority, £12 M, is produced by the Drugs and Alcohol services (Table 6).

**Table 6:** Return on investment for five commissioned public health services (PVM isn't calculated where there is a net present budget impact).

**Commissioned programmes**	**Costs**	**Total programme benefit**		**Return on investment**		**Benefit-cost ratio**	**Payback period**
	**(£m)**	**Public (£m)**	**Fiscal (£m)**	**Public (£)**	**Fiscal (£)**	**PVM**	**PP**
Live well dorset	1	5	1	6.81	1.37	Not applicable	8 years
Drug and alcohol	4	12	5	2.70	1.14	Not applicable	9 years
Dorset smoke stop	1	2	0.2	2.39	0.28	1.92	–
Health checks	1	1	0.1	1.53	0.15	0.62	–
Sexual health	7	10	4	1.37	0.54	0.79	–
Total	14	30	10	2.16	0.74	4.54	

The commissioned public health services provide an overall return on investment of £2.51 for every £1.00 invested. Additionally, the five service areas, composed of 13 outcome areas, show a clear ranking of public benefit return on investment from LiveWell Dorset at £6.81 through to sexual health services that provide an estimated public benefit of only £1.33 for £1 invested (Table 6).

### Fiscal Return on Investment

The estimated 10-year fiscal return from the five commissioned programmes is approximately £10 M in direct savings to other public service organizations across the Dorset system. The fiscal return on investment again shows that that LiveWell Dorset, £1.37, and the Drugs and Alcohol services, £1.14, provide the greatest return on every £1 invested, but there is little to distinguish between the other three service areas whose fiscal returns are in the £0.15–£0.52 range (Table 6).

**Figure 2:**
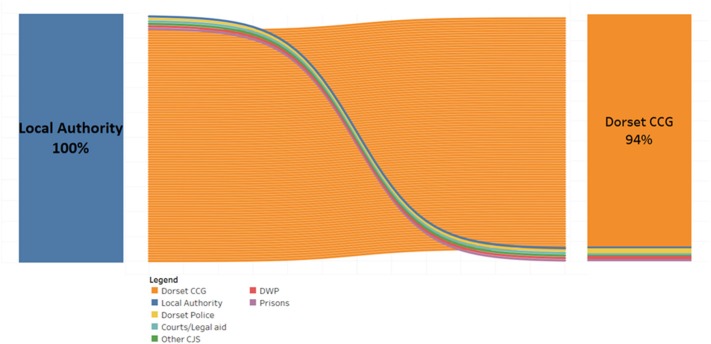
The organizations that benefit from public health intervention in Dorset.

The fiscal return-on-investment estimates the direct financial impact on government agencies and delivery partners. It indicates the total impact on the budget of organizations delivering or benefiting from the project over the 10 years we have modeled the project's benefits. The Dorset CCG benefits the most from these commissioned services, with local authorities benefiting significantly less. The CCG receives most of their benefit from the public health commissioned Drug and Alcohol services, while the local authorities receive most of their benefit from LiveWell Dorset (Figure 2).

The standard Green Book discount rate of 3.5% accounts the depreciated value of a return or cost some years in the future. These totalled produce Net Present Values (NPV). These are a measure of the additional value created by implementing the programmes. The Net Present Value over the 10-year period is £18m, if all benefits are estimating a fiscal return.

### Public Value for Money

The model calculates an additional metric, for intervention programmes without a net positive fiscal impact, called the Public Value for Money (PVM). The PVM metric attempts to qualify interventions where there isn't a net positive fiscal impact and illustrates that in the case of the Smoke Stop programme there is still a public value after taking this position into account in terms of its public value cost-benefit ratio of 1.92. The sexual health and Health Checks programmes, however, even appears to fail the PVM test, returning a negative benefit-cost ratio of 0.79 and 0.62, respectively. This is a particularly small value because programme costs are relatively high; it is negative because the programme costs exceed even the estimated public benefit.

## Discussion

### Main Finding of This Study

LWD and Drugs and alcohol services provide the best return on our public health investments. The fiscal benefits of these interventions flow to the NHS through our CCG.

### What Is Already Known on This Topic

Summarized evidence relating to approximately 200 public health interventions, including areas of smoking, alcohol and physical activity, shows that the vast majority of these interventions are highly cost-effective, and in most cases are far below the recommended NICE threshold of £20,000 per Quality-Adjusted Life Year (QALY) (22). Additionally, a recent systematic review suggests that local and national public health interventions are highly cost saving, demonstrating a median return on investment of £14 for every £1 invested in public health that indicates cuts to public health are short sighted with substantial opportunity costs (23).

### What This Study Adds

We provide a clear ranking of return on investment from five public health programmes that we commission. We approached this work asking the question “is cost-benefit analysis helpful?” to our decision-making processes and we think it “can be” (we have only recently begun generating these data, so we do not have the ability to reflect on decisions already supported through CBA) in the following areas:

- Separating the “wheat from the chaff”—our CBA immediately identifies a qualitative difference between the Live Well Dorset services, our Drugs and Alcohol programmes and three other commissioned services.
- It focussing our limited intelligence resources on filling identified knowledge gaps, i.e., are we not understanding some significant benefits delivered by the sexual health programme, or are these benefits just not worth the cost?
- Which organizations benefit fiscally from Public Health Dorset programmes? In an integrated “whole of system” approach this needs to be part of the discussion.

Our work has generated significant interest more widely in our local system, where many budgets face similar challenges. As with the public health budget, many budgets within local authorities evolved over long periods of time with myriad factors having played a non-transparent role in their current composition and distribution. Attempts to normalize or “zero base” budgets are complex and difficult and as a result are rarely attempted. Departmental and directorate budgets are jealously guarded and attempts to rationalize spend across differing areas of authority activity generate a lot of noise but little light.

### Limitations of This Study

#### Marginal Analysis

The cost-benefit analysis presented here is a first step toward providing local public health decision-making with consistent indicators of the value of their spending on commissioned services. The next step, which is equally important, is to undertake a comprehensive sensitivity analysis of these data, to determine what the marginal advantage of spending more in areas that are indicatively high public value for money and less on areas with low public value for money—will the additionally or marginal change in spending be as valuable?

#### Sense Checking Assumptions

One of the strengths of cost-benefit analysis is that with few exceptions it is completely transparent through the provision of a few tables of internal parameters used in the calculation. We often do not have the resources to thoroughly research and sense check these data, but in the case of our sexual health programme, we may have failed to capture all the public benefits that the programme delivers and/or there are unaccounted for implications. Indeed, this is probably for all programmes modeled, with the acknowledgment that our assumptions are transparent and can be used as the basis for initiating system-wide discussions. “These results do not accurately reflect the differing demographics of the risk populations considered by programme within the Dorset population, and several programmes will have overlapping risk populations, as such the results should be viewed in this light.”

#### Unaccounted for Implications

for example, are there disadvantaged groups that might be receiving disproportionate support from some of the services with lower return on investment, implying that disinvestment will increase health inequality? Are there epidemiological considerations concerning these investments, such as the reduction of epidemic risk that sexual health services might provide and into which we will have to delve more deeply?

## Conclusion

Amongst the five major public health programmes examined, Live Well Dorset represents, by a significant margin, the best return on investment, with Drug and Alcohol commissioned services also providing a very good return on investment, relative to the other commissioned public health programmes. If these measures were to influence decision-making within local public health spending, it would argue that expenditure should be transferred from commissioned sexual health services into Live Well Dorset and Drug and Alcohol services.

However, we have additional work to do in understanding the marginal benefits from increasing the budgets of these two programmes, as well as the health outcome impacts resulting from defunding other areas of work. The alternative to this approach in an era of shrinking budgets is to treat all programmes similarly and “salami slice” what we can off each programme, each year, until the programme can no longer be sustained in any form. A further discussion and debate will be had around the poorer returns on investment, Health Checks and Sexual Health programmes, in terms of their nationally mandated status. This constrains local decision-making in terms of where limited resources are best invited in any particular locality.

## Author Contributions

LR and CS contributed conception and design of the study. LR performed the statistical analysis and wrote the first draft of the manuscript. LR, CS, and DP wrote sections of the manuscript. All authors contributed to manuscript revision, read and approved the submitted version.

### Conflict of Interest Statement

The authors declare that the research was conducted in the absence of any commercial or financial relationships that could be construed as a potential conflict of interest.
